# Saturation-Recovery Myocardial T_1_-Mapping during Systole: Accurate and Robust Quantification in the Presence of Arrhythmia

**DOI:** 10.1038/s41598-018-23506-z

**Published:** 2018-03-27

**Authors:** Nadja M. Meßner, Johannes Budjan, Dirk Loßnitzer, Theano Papavassiliu, Lothar R. Schad, Sebastian Weingärtner, Frank G. Zöllner

**Affiliations:** 1 Computer Assisted Clinical Medicine, Medical Faculty Mannheim, Heidelberg University, Mannheim, Germany; 2 DZHK (German Centre for Cardiovascular Research) partner site Heidelberg/Mannheim, Mannheim, Germany; 3 Department of Clinical Radiology and Nuclear Medicine, University Medical Center Mannheim, Medical Faculty Mannheim, Heidelberg University, Mannheim, Germany; 4 1st Department of Medicine Cardiology, University Medical Center Mannheim, Medical Faculty Mannheim, Heidelberg University, Mannheim, Germany; 5 Department of Electrical and Computer Engineering, University of Minnesota, Minneapolis, MN United States; 6 Center for Magnetic Resonance Research, University of Minnesota, Minneapolis, MN United States

## Abstract

Myocardial T_1_-mapping, a cardiac magnetic resonance imaging technique, facilitates a quantitative measure of fibrosis which is linked to numerous cardiovascular symptoms. To overcome the problems of common techniques, including lack of accuracy and robustness against partial-voluming and heart-rate variability, we introduce a systolic saturation-recovery T_1_-mapping method. The Saturation-Pulse Prepared Heart-rate independent Inversion-Recovery (SAPPHIRE) T_1_-mapping method was modified to enable imaging during systole. Phantom measurements were used to evaluate the insensitivity of systolic T_1_-mapping towards heart-rate variability. *In-vivo* feasibility and accuracy were demonstrated in ten healthy volunteers with native and post-contrast T_1_-mappping during systole and diastole. To show benefits in the presence of RR-variability, six arrhythmic patients underwent native T_1_-mapping. Resulting systolic SAPPHIRE T_1_-values showed no dependence on arrhythmia in phantom (CoV < 1%). *In-vivo*, significantly lower T_1_ (1563 ± 56 ms, precision: 84.8 ms) and ECV-values (0.20 ± 0.03) than during diastole (T_1_ = 1580 ± 62 ms, p = 0.0124; precision: 60.2 ms, p = 0.03; ECV = 0.21 ± 0.03, p = 0.0098) were measured, with a strong correlation of systolic and diastolic T_1_ (r = 0.89). In patients, mis-triggering-induced motion caused significant imaging artifacts in diastolic T_1_-maps, whereas systolic T_1_-maps displayed resilience to arrythmia. In conclusion, the proposed method enables saturation-recovery T_1_-mapping during systole, providing increased robustness against partial-voluming compared to diastolic imaging, for the benefit of T_1_-measurements in arrhythmic patients.

## Introduction

Cardiac magnetic resonance imaging enables the assessment of cardiac anatomy and function and the detection of myocardial fibrosis, which is linked to numerous cardiovascular adverse cardiovascular events like heart failure, arrhythmia and sudden cardiac death^1^. In addition to late gadolinium enhancement as a robust standard for focal fibrosis, even diffuse cardiac pathologies can now be assessed with T_1_-mapping, the non-invasive alternative to biopsy^2^.

T_1_-maps are obtained by acquiring multiple sample points on a longitudinal magnetization recovery curve after magnetization preparation. Imaging at the same cardiac phase yields co-registered images, henceforth referred to as base-images, and pixel-wise curve fitting allows for spatially resolved quantification of T_1_^3^. Pre- and post-contrast T_1_-mapping further permit estimation of the extracellular volume (ECV) fraction. Both biomarkers have shown to be predictors of mortality in cardiovascular disease and bear promise for risk stratification^4^.

However, partial-volume effects at the interface between myocardium and blood-pool corrupt quantification accuracy and impair reproducibility in T_1_-mapping^5,6^. Hence, imaging during systole, exploiting increased myocardial thickness, has recently been proposed^7^ and demonstrated improved quality in patients with atrial fibrillation^8^. However, the modified Look-Locker inversion recovery (IR) technique (MOLLI)^3^ was used in these studies, which is known to underestimate T_1_-values^9^, and to be affected by the patient’s heart-rate^10^ and various imaging parameters^11^. Alternatively, saturation-recovery (SR) T_1_-mapping, as realized by the Saturation-recovery single-shot acquisition (SASHA)^12^ technique, provides more accurate T_1_-values, for the trade-off against reduced precision. A hybrid version of IR and SR, called Saturation-Pulse Prepared Heart-rate independent Inversion-Recovery (SAPPHIRE)^10^, has been proposed to enable accurate T_1_-quantification with increased precision compared to saturation-recovery only. However, systolic imaging cannot be performed with the previously proposed SASHA and SAPPHIRE sequences, as the preparation time between R-wave detection and systolic imaging is insufficient for magnetization recovery, leading to low SNR in base-images and compromising T_1_-fit quality.

The purpose of this study is to develop a method for systolic saturation-recovery T_1_-mapping and ECV-calculation at 3T and to provide robust image quality in patients suffering from arrhythmia.

## Materials and Methods

### Numerical Simulation

The influence of mis-triggering artifacts on conventional SAPPHIRE T_1_-maps was simulated in a 650 × 650 pixel numerical phantom, representing a mid-ventricular short-axis of the left-ventricular (LV) myocardium, left- and right-ventricular (RV) blood, and epicardial fat (T_1_ = 1578; 2048; 382 ms, respectively)^13^. Systolic images were designed with reduced LV diameter (70%) and increased myocardial thickness (140%) (16). Ten base-images were generated by assigning signal values to the compartments calculated from Bloch simulations of the SAPPHIRE signal equation (inversion-times TI = [10000;805;113;211;309;407;505;603;701;799 ms]; trigger delay TD = 805 ms). Additive Gaussian noise was subsequently added (SNR = 60). Mis-triggering effects were mimicked by replacing diastolic by systolic base-images in randomized order. The share of systolic images was calculated in terms of four arrhythmia factors, with standard deviations of 0%, 30%, 60%, and 70% of the mean RR length (667 ms), based on previous literature^14^. Each variant was simulated 50 times and analyzed for mean T_1_ (accuracy) and standard-deviation across repetitions (precision).

### Systolic SAPPHIRE Sequence Design

**Figure 1:**
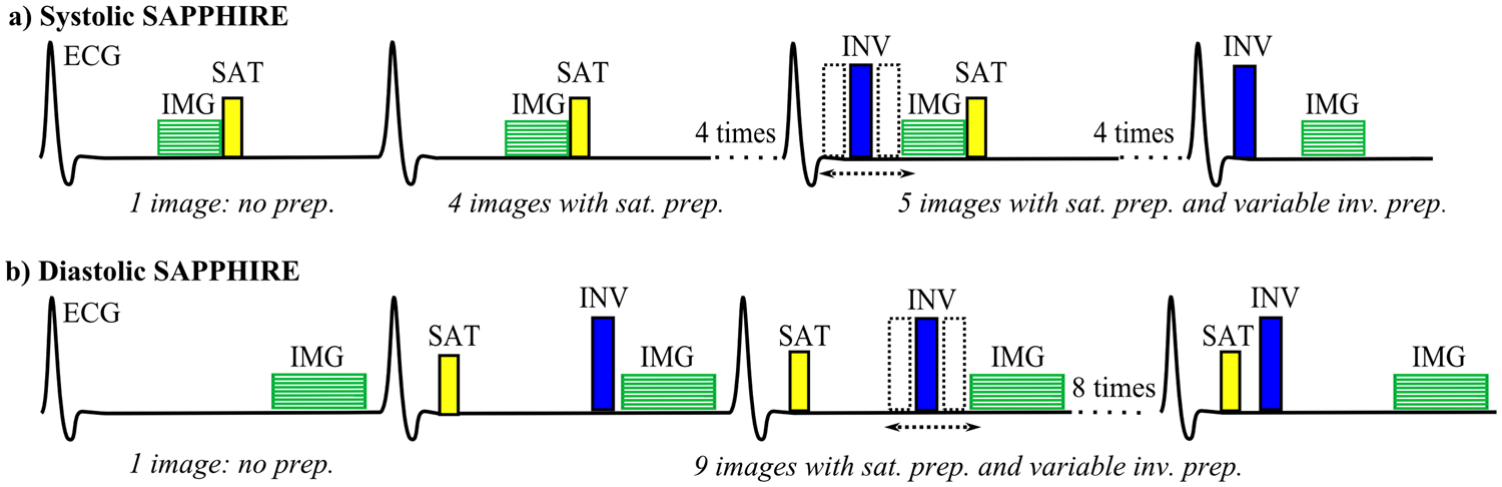
Sequence diagrams of the systolic (**a**) and diastolic (**b**) SAPPHIRE T_1_-mapping sequence with ten readouts, as used in phantom and in healthy volunteers. Systolic T_1_-mapping starts with one image (IMG) without preparation, the following four images (seven images in patients) are preceded by a saturation pulse (SAT) in the heart beat before imaging, directly after the previous image acquisition. The remaining images (four in healthy, seven in patients) are acquired with an additional inversion-pulse (INV) with variable delay after the R-wave. (**b**) In diastolic (conventional) SAPPHIRE, the first image acquisition is also performed without magnetization preparation. However, for the remaining images, both the saturation- and the inversion-pulse are played within the same heartbeat before image acquisition. The image acquisition window is longer compared with systole.

The proposed T_1_-mapping variant consists of a hybrid saturation/inversion-recovery magnetization preparation and 10 ECG-triggered readouts (in patients 15 readouts, respectively). The average scan time in healthy subjects was 10 sec, in patients 12 sec, trading-off the higher number of images against the faster heart rate. In systole, the limited time between the R-wave and imaging yields insufficient signal for conventional SR T_1_-mapping. To overcome this, the saturation-pulse is played in the preceding heartbeat after imaging (Fig. 1). The first image is acquired without preparation, yielding full recovery. Four images are acquired with saturation-preparation (WET module^13^) only, to obtain maximal recovery. Five images are additionally prepared with an inversion-pulse (adiabatic full passage tan/tanh pulse^15^) between the R-wave and image acquisition, with linearly spread inversion-times.

### Data Acquisition

Images were acquired at a 3T MRI scanner (Magnetom Skyra; Siemens Healthcare, Erlangen, Germany) with a 30-channel receiver coil array. For systolic acquisition, a single-shot balanced Steady-State Free Precession (bSSFP) readout was used with the following parameters (“systolic parameter set”): TR/TE/α = 2.6 ms/1.0 ms/35°, in-plane resolution = 1.2 × 1.2 mm^2^, slice-thickness = 6 mm, field-of-view = 350 × 263 mm^2^, bandwidth = 1240 Hz/px, #k-space-lines = 57, linear profile-ordering, startup-pulses = 5 Kaiser-Bessel, GRAPPA-factor = 3. Diastolic T_1_-maps were acquired with longer acquisition windows using the following parameters (“diastolic parameter set): TR/TE/α = 2.6 ms/1.0 ms/35°, in-plane resolution = 1.7 × 1.7 mm^2^, slice-thickness = 8 mm, field-of-view = 440 × 375 mm^2^, bandwidth = 1085 Hz/px, #k-space-lines = 139, linear profile-ordering, startup-pulses = 5 Kaiser-Bessel, GRAPPA-factor = 2. Modified Look-Locker Inversion Recovery (MOLLI)^3^ T_1_-maps were acquired in the 5(3)3 scheme with the diastolic parameter set for both diastolic and systolic acquisition. For the latter, the acquisition window was shortened and shifted towards the systole.

### Phantom Experiments

To study the influence of arrhythmia on T_1_ in systole, scans were performed in seven vials containing agarose gel, doped with various concentrations of a gadoterate meglumine contrast agent (Dotarem; Guerbet, Aulnay-sous-Bois, France). Heart-rate variability was simulated by a pause of random duration before the R-wave, resulting in RR-interval standard-deviations of 0, 200, 400, and 500 ms, respectively.

### *In Vivo* Experiments

This prospective study was approved by the Institutional Review Board II, Medical Faculty Mannheim, Germany, and written informed consent was obtained from all volunteers. We hereby confirm that all experiments were performed in accordance with relevant guidelines and regulations.

As a preliminary substudy, five healthy volunteers (3 f, 26 ± 3 y) underwent native T_1_-mapping with six different variants: (a) diastolic MOLLI, (b) systolic MOLLI, (c) diastolic SAPPHIRE with ‘diastolic parameter set’, (d) systolic SAPPHIRE with ‘systolic parameter set’, (e) systolic SAPPHIRE with ‘diastolic parameter set’ and shortened trigger delay and (f) systolic SAPPHIRE with ‘systolic paramter set’ and an increased number of base images (15 images).

Ten healthy volunteers (5 m, 25 ± 4 y) underwent T_1_-mapping before and 15 min after injection of 0.2 mmol/kg Dotarem. T_1_-maps were acquired in three short-axis slices during systole with the proposed method and during diastole with conventional SAPPHIRE. Timing for systolic acquisition was visually determined from short-axis cine images. To avoid a systematic influence of contrast agent washout, sequence and slice orders were randomized. Blood samples were drawn to measure blood hematocrit for ECV calculations.

Six patients (4 m, 52 ± 19 y) underwent native T_1_-mapping in a mid-ventricular short-axis slice with systolic and diastolic SAPPHIRE. They partially displayed substantial arrhythmia during the scan, as can be seen in Table 1 on patient characteristics.

**Table 1:** Characteristics of the six arrhythmic patients (4 m, 52 ± 19 y) including their indications for cardiac MRI and their variability in RR length.

Patient N^o^	indication	Variability in RR length in ms		
		RR_mean_ ± std	RR_min_	RR_max_
1	ischemic cardiomyopathy, moderate reduced LV-function and a high burden of premature ventricular contraction	816 ± 134	613	1068
2	dilated cardiomyopathy with mild LV-dysfunction	989 ± 275	665	1595
3	coronary fistula with normal LV-function, but premature ventricular contration and bigeminy	781 ± 136	618	973
4	multifocal premature ventricular contractions on the Holter ECG	1226 ± 69	1075	1308
5	multifocal premature ventricular contractions on the Holter ECG	1514 ± 426	890	2375
6	coronary artery disease, atrial fibrillation and moderate reduced LV-function	745 ± 224	560	1333

### Data Analysis and Statistics

MATLAB R2014a (Mathworks; Natick, MA, USA) was used for image evaluation and statistics. For T_1_-estimation, a 3-parameter least-squares fit to the T_1_-recovery curve was performed. T_1_-map quality in numerical simulation was evaluated with standard-deviation maps. In phantom, T_1_-times were analyzed in manually drawn ROIs. *In vivo*, pixel-wise fitting was performed to generate T_1_-maps, followed by segmentation according to the AHA-16-segment-model^16^, with estimation of T_1_ and ECV as mean per segment. Precision was defined as the intra-segment variation in terms of standard-deviation. Blood T_1_-times were evaluated from manually drawn ROIs in the LV-blood-pool. Diastolic T_1_-maps were estimated with magnitude-images after polarity restoration^3^. Phase-sensitive fitting was used in systole by subtracting the phase of the non-magnetization-prepared base-image from the remaining images. The resulting phase difference was thresholded $(|{\rm{\Delta }}\phi | > \pi /2\,{\rm{a}}{\rm{n}}{\rm{d}}\,|{\rm{\Delta }}\phi |\le \pi /2\,)$ after phase unwrapping^17^ to yield a signal polarity map, which was finally multiplied to all systolic base-images.

To assess the amount of myocardial tissue suited for T_1_-evaluation, myocardial thickness was evaluated in healthy volunteers as the area between manually drawn LV endo- and epicardial borders in systole and diastole and in all slices. To estimate the effect of partial-voluming, the full-width-at-half-maximum (FWHM) of T_1_-intensity line profiles from the LV to the RV blood-pool across the center of the septum was determined. The lack of a clearly depicted RV blood-pool in apical slices prevented their inclusion in the FWHM-analysis.

In phantom, a coefficient of variation (CoV) was calculated as the ratio of T_1_-standard-deviation to mean T_1_. For statistical comparison of systole and diastole *in vivo*, T_1_- and ECV-values were studied with a paired student’s t-test, and T_1_-precision with a Mann–Whitney-U-test (significance for p < 0.05).

## Results

**Figure 2:**
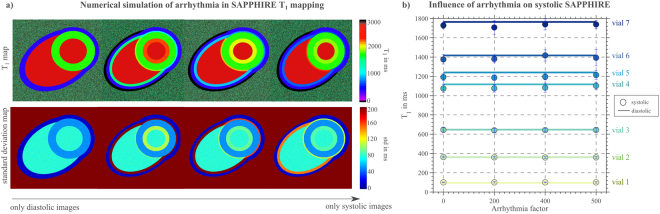
Influence of arrhythmia studied in (**a**) a numerical simulation and (**b**) in phantom measurements. (**a**) Simulated influence of arrhythmia on diastolic T_1_-map quality is shown on a model mid-ventricular short-axis view and the corresponding standard deviation map. Blurring at the endo- and epicardial borders increased with increasing number of mis-triggered base-images. (**b**) Influence of simulated heart-rate variations on SAPPHIRE T_1_-values for seven phantom vials covering a broad T_1_-range. Circles indicate the T_1_-results with systolic SAPPHIRE for four different arrhythmia factors (defined as the standard deviation from the Gaussian distribution of a mean RR-length of 1125 ms). Reference diastolic T_1_-values are given as solid lines. No major influence of arrhythmia on systolic T_1_-values was found.

Numerical simulations revealed a degrading effect of mis-triggering on conventional SAPPHIRE T_1_-map quality in terms of blurring at myocardial borders, with increasing severity at higher degrees of arrhythmia (Fig. 2a). Accordingly, standard-deviation maps showed higher variation in border regions.

In phantom, systolic SAPPHIRE T_1_-mapping results were independent of arrhythmia, as shown in Fig. 2b, yielding a CoV <1%.

**Figure 3:**
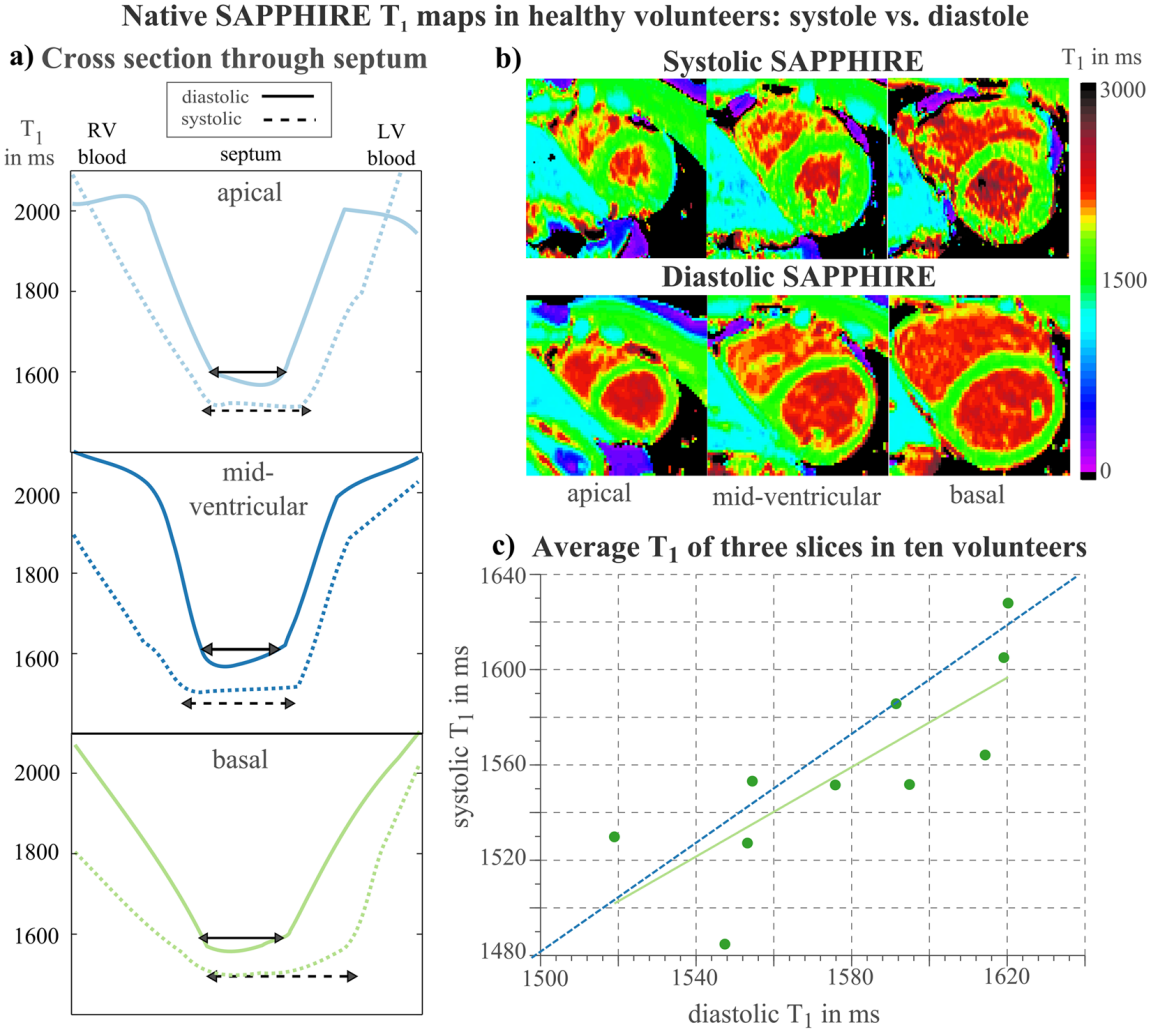
Reduction of partial-volume effects by higher myocardial thickness in systole. (**a**) Cross-sections through the LV septum in apical, mid-ventricular and basal short axis T_1_-maps, acquired with systolic and diastolic SAPPHIRE. The cross-sections through diastolic T_1_-maps (solid lines) show strongly elevated T_1_-times at endo- and epicardial borders, whereas in systole (dotted), this effect is reduced. Corresponding T_1_-maps of a healthy volunteer (m, 23 y) are shown in (**b**). The apparent myocardial thickness is clearly higher in systole compared to diastole, so more myocardial voxels can be included into T_1_-estimation without risking an elevation of T_1_-times by partial-volume effects from the highly intense blood-pool. (**c**) Correlation of systolic and diastolic native T_1_ in ten healthy subjects, each point indicating the average over the three slices in each subject, revealing a strong positive correlation of the two methods. The identity line is indicated in dashed blue, the solid green line represents best fit.

*In vivo* T_1_-mapping was successfully performed in all healthy subjects. bSSFP banding artifacts led to the exclusion of 26 out of 2080 segments (1.3%). Partial-volume effects showed a higher impact on diastolic than on systolic T_1_-maps, as shown by a LV septal cross-section plot in Fig. 3a. In diastole, T_1_-times at septal borders are clearly elevated towards the blood-pools, leaving only the inner region of the septum unaffected of partial-voluming. In systolic T_1_-maps, however, larger plateaus for the estimation of myocardial T_1_-times are available, as reflected by the significant increase in mean FWHM (mid-ventricular: 167 ± 37%; basal: 224 ± 89%) compared to diastole. Corresponding native T_1_-maps of a healthy volunteer, where the increase in myocardial thickness from diastole to systole is clearly depicted, are shown in Fig. 3b. In average over all vounteers, the measured increase of myocardial thickness during systole is significant (apical: 255 ± 85%; mid-ventricular: 254 ± 63%; basal: 209 ± 40%; p < 10^−4^). Systolic and diastolic T_1_ display strong positive correlation (r = 0.89) **(**Fig. 3c**)**.

**Figure 4:**
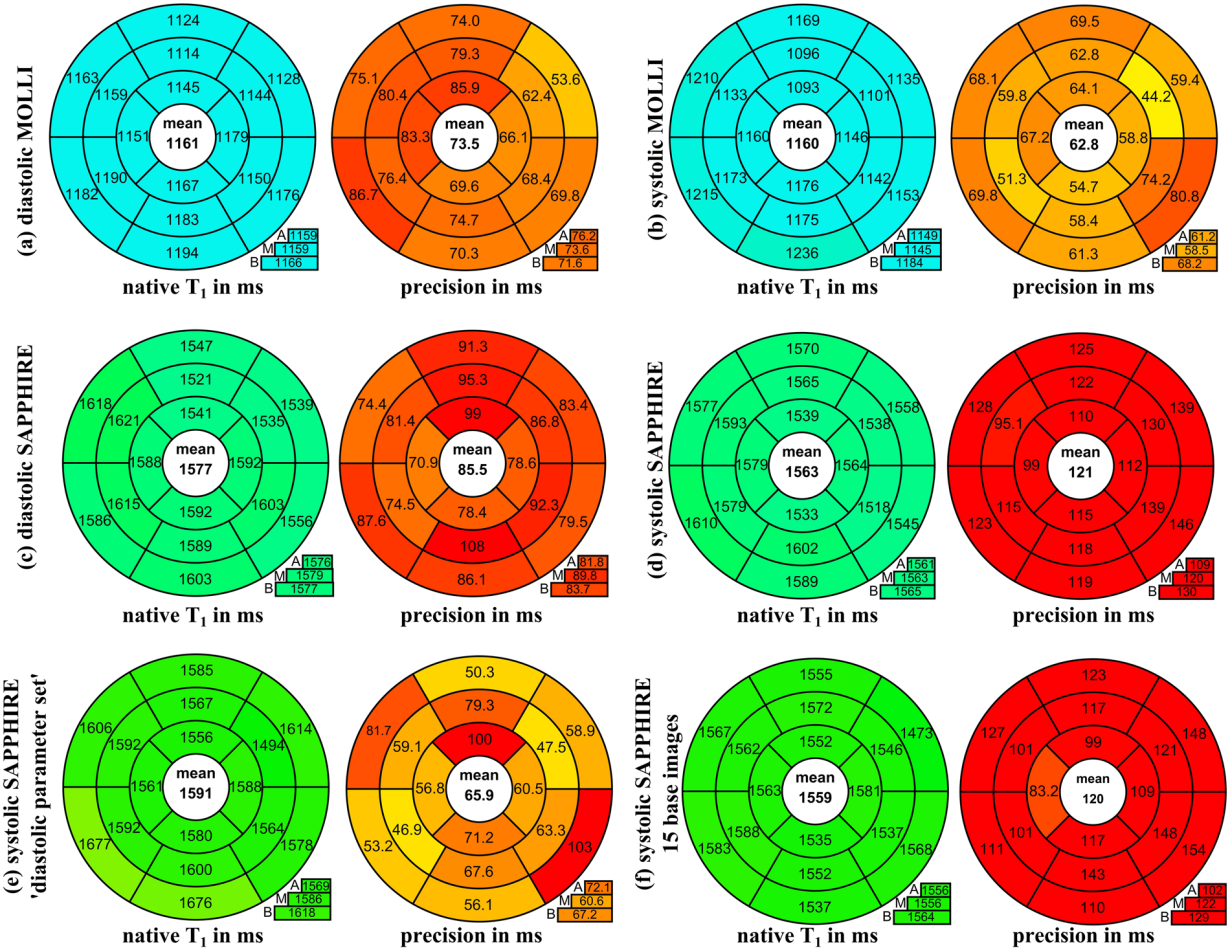
Indicative substudy for the comparison of MOLLI and SAPPHIRE sequence variants. Mean values of five healthy volunteers (3 f, 26 ± 3 y) in three short-axis slices (A = apical, M = mid-ventricular, B = basal) are displayed as bullseye plots (AHA-16-segment-model) for native myocardial T_1_-times and T_1_-time precision. They have been acquired with different sequence variants of the MOLLI and the SAPPHIRE T_1_-mapping method. The average across all segments is given in the bullseye centers, slice averages in the boxes below.

The results of a preliminary comparison of different T_1_-mapping results in five healthy volunteers are visualized in Fig. 4. Systolic MOLLI T_1_-times (T_1_ = 1160 ± 55 ms, precision:62.8 ms) are significantly lower than systolic SAPPHIRE T_1_-times (T_1_ = 1563 ± 40 ms, precision:121 ms) (p < 10^−6^), which is in accordance with previous literature^13^. No significant difference between MOLLI T_1_-times acquired in diastole (T_1_ = 1161 ± 40 ms, precision:73.5) and in systole (T_1_ = 1160 ± 55 ms, precision:62.8 ms) was found (p = 0.8) in this initial cohort. Diastolic SAPPHIRE T_1_-times (T_1_ = 1577 ms ± 48 ms, precision:85.5 ms) are significantly higher than systolic SAPPHIRE T_1_-times (T_1_ = 1563 ± 40 ms, precision:121 ms) when the optimized ‘systolic parameter set’ is used for the latter (p < 10^−3^). Systolic SAPPHIRE T_1_-times acquired with the ‘diastolic parameter set’ were higher than the previous two (T_1_ = 1591 ± 84 ms, precision:65.9) in average. However, due to the limited cohort size no significance was found in the differences between systolic and diastolic parameter sets, despite this major difference in the average value. Systolic SAPPHIRE with 15 base images (T_1_ = 1559 ± 40 ms, precision:120 ms), yielded T_1_-times comparable to the systolic SAPPHIRE with 10 base images in terms of T_1_-time and precision (T_1_ = 1563 ± 40 ms, precision:121 ms) (p = 0.2).

**Figure 5:**
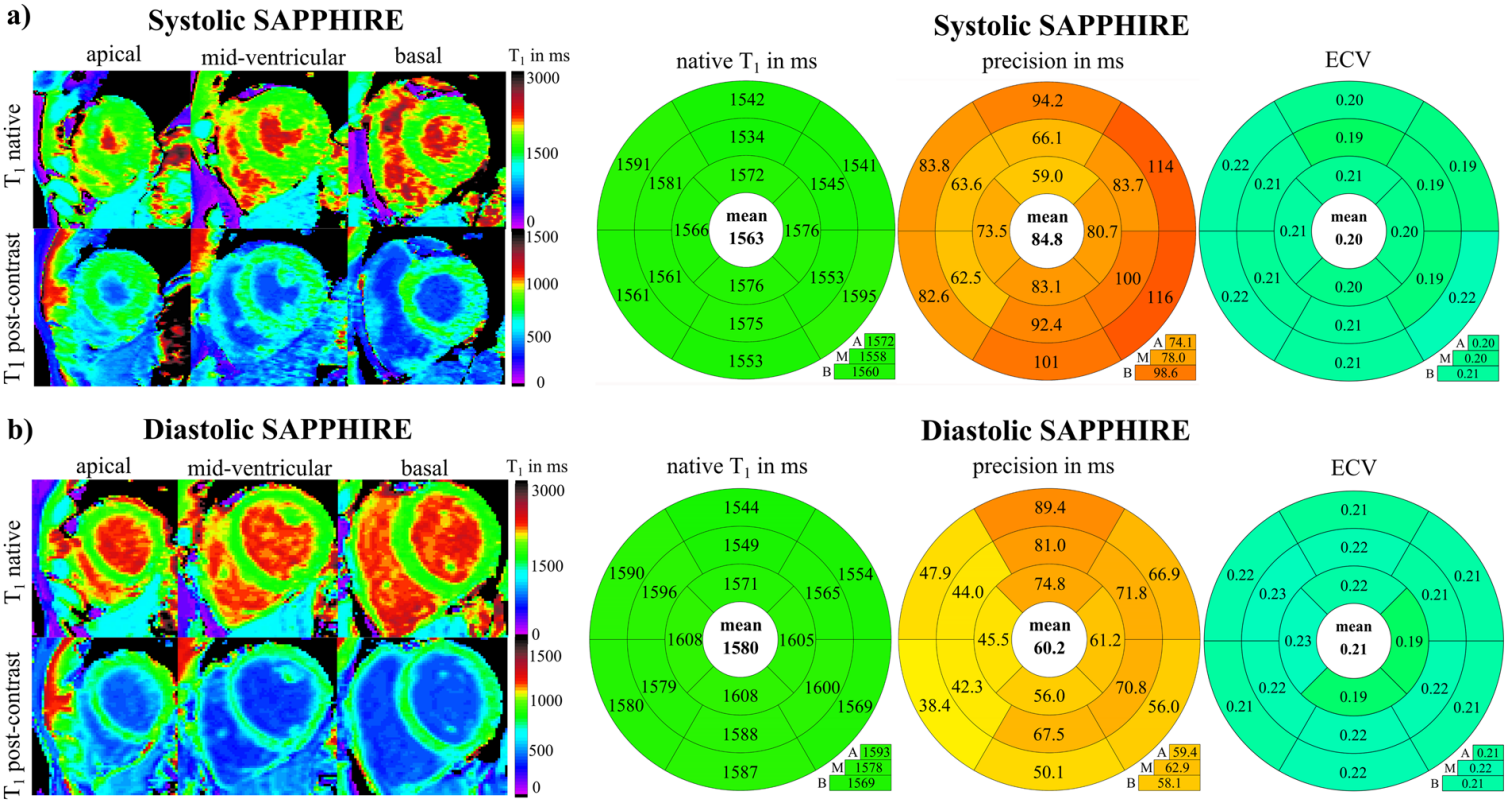
The left side shows native and post-contrast T_1_-maps of a healthy volunteer (m, 35 y), acquired with systolic (**a**) and diastolic (**b**) T_1_-mapping. T_1_-map quality is visually high in short axis apical (left), mid-ventricular (middle) and basal (right) slices. Mean values of ten healthy volunteers (5 m, 25 ± 4 y) in three short-axis slices (A = apical, M = mid-ventricular, B = basal) are displayed as bullseye plots (AHA-16-segment-model) for native myocardial T_1_- times, T_1_-time precision and ECV, acquired with the systolic SAPPHIRE technique (**a**) and the diastolic SAPPHIRE technique (**b**). The average across all segments is given in the bullseye centers, slice averages in the boxes below.

Figure 5 compares *in-vivo* systolic (a) and diastolic (b) pre- and post-contrast SAPPHIRE T_1_-maps in ten healthy volunteers. Excellent T_1_-map quality, a high contrast and homogeneous T_1_-values, indicating high precision, were achieved. Bullseye-plots on the right show that systolic SAPPHIRE T_1_-times (1563 ± 56 ms, precision: 84.8 ms) are significantly lower than diastolic T_1_-times (1580 ± 62 ms, precision: 60.2 ms) (T_1_: p = 0.0124; precision: p = 0.0098). Accordingly, systolic and diastolic ECV-values (0.20 ± 0.03/0.21 ± 0.03) show significant differences (p = 0.03).

**Figure 6:**
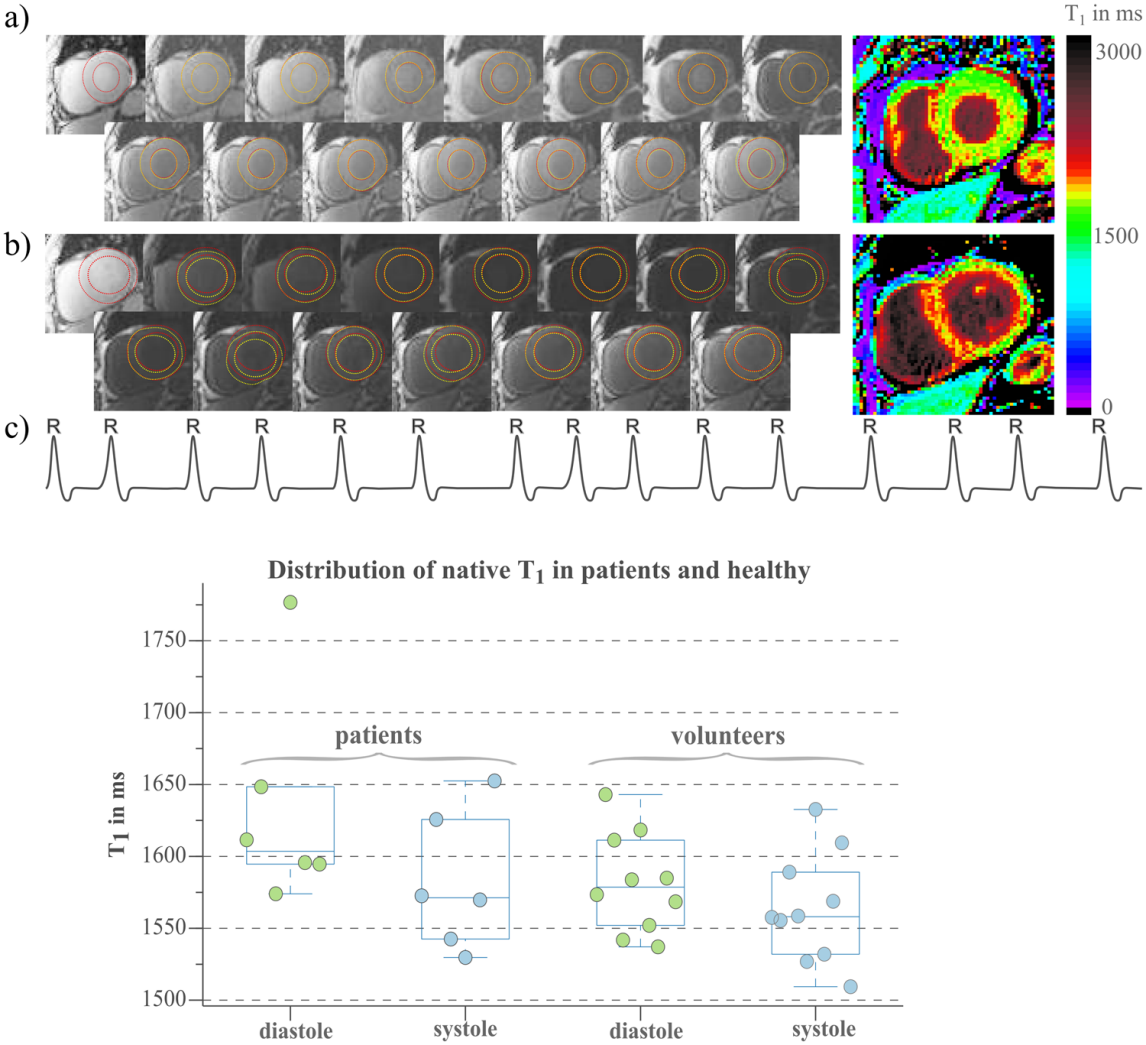
SAPPHIRE T_1_-mapping data in systolic (**a**) and diastolic (**b**) acquisition of a patient (m, 73 y) suffering from arrhythmia. Left ventricular myocardial borders are delineated in red in all 15 recovery images for the myocardial borders of the first cardiac frame, in yellow for all the following frames. In systole, the borders accord with the first frame, which reflects in less artifacts on the T_1_-map on the right. The detected 15 RR-lengths during that measurement are depicted below (**c**) to visualize the arrhythmia (variability in RR length (RR mean ± std) = 816 ± 134 ms; min: 613 ms; max: 1068 ms). (**d**) Boxplots showing native T_1_-estimations with systolic and diastolic SAPPHIRE of six patients (4 m, 52 ± 19 y) suffering from arrhythmia and ten healthy volunteers (5 m, 25 ± 4 y). Superimposed scattered data points indicate mean T_1_ per subject.

Figure 6 depicts all base-images and corresponding T_1_-maps from a patient suffering from arrhythmia during the scan (c). Due to mis-triggering-induced motion between the base-images, significant artifacts are visible with diastolic SAPPHIRE (b), extending throughout the LV-myocardium and being most severe in the septum. No such artifacts are observed with systolic SAPPHIRE (a), displaying resilience to arrythmia. For both systole and diastole, patient T_1_ was clearly elevated compared to T_1_ in healthy, as shown in (d). In patients, mean mid-ventricular systolic T_1_-values (1582 ± 48 ms) are significantly lower than distolic T_1_ (1633 ± 74 ms) (p = 0.0311).

## Discussion

In this study, the saturation-recovery T_1_-mapping sequence SAPPHIRE was modified to robustly measure myocardial T_1_ during systole in arrhythmic patients. Excellent T_1_-map quality was achieved in healthy volunteers and arrhythmic patients, despite the short and early systolic acquisition window. In healthy, a significant difference between systolic and diastolic T_1_- and ECV-values was found, most likely to be explained by reduced partial-volume effects in systole.

Unlike inversion-recovery T_1_-mapping techniques, SAPPHIRE has predefined delays after saturation and inversion, enabling robust and accurate T_1_-mapping across subjects with different heart-rates. However, numerical simulations revealed a degrading effect of mis-triggering on diastolic T_1_-mapping. Accordingly, T_1_-mapping in arrhythmic patients showed that major variations in diastolic duration led to artifacts. The problem stems from the single fixed trigger-time for multiple image acquisitions: If the diastolic phase shortens significantly during imaging, the trigger-time extends beyond the occurrence of the next R-wave. The systolic phase, however, is not subject to shortening in all common arrhythmias^18^, enabling a stationary time for imaging with a lower risk of mis-triggering. However, due to the brevity of the quiescent period and the high myocardial mobility, systolic T_1_-mapping is performed with shorter acquisition windows to minimize temporal blurring. Diastolic methods commonly employ acquisition windows up to ~360 ms, which extends far beyond systolic quiescence. For systole, a higher GRAPPA-factor (GRAPPA = 3 versus 2) and an increased bandwidth were chosen to reach a temporal resolution of ~160 ms. This trade-off led to decreased precision in systolic T_1_-maps of healthy volunteers_._ Future studies will focus on employing advanced acceleration techniques^19,20^, potentially exploiting the interdependence between the baseline images^21^, in order to mitigate this loss in precision. In systole, moreover, a smaller slice thickness of 6 mm (versus 8 mm in diastole) was chosen to avoid partial volume effects with the high signal from the adjacent blood pool, which is particularly important in systolic imaging as the cardiac long axis is substantially shortened during the contraction. A preliminary comparison in five healthy volunteers between the proposed systolic SAPPHIRE method and the systolic SAPPHIRE method with the ‘diastolic parameter set’ and only one modified parameter (imaging phase) led to a higher mean value in the latter case. However, due to the small sample size of five volunteers, no statistically significant difference could be shown.

An alternative to mitigate motion artifacts from mis-triggering is image co-registration. However, substantial contrast variations between the base-images hamper the use of conventional methods. Dedicated registration algorithms based on image synthetization^22^ or contrast-variation-adapted optical flow^23^ have been proposed to improve parameter map quality and decrease spatial variability by alleviating breathing motion effects^24^, which are mostly translational. Mis-triggering and imaging during systole, however, are non-translational. Moreover, saturation-recovery yields, compared with inversion-recovery, low baseline SNR, further reducing the effectiveness of registration algorithms^13^. Imaging during systole, on the other hand, robustly ensures image co-registration regardless of baseline SNR and without extensive post-processing with dedicated non-rigid contrast-adapted registration methods.

Usually, to allow fitting of magnitude-images to a parameter model with negative values, base-images are sorted by their TI and point-wise successive flipping of polarity is performed until best fit is reached. In systolic SAPPHIRE, both TI and the saturation-time TS are variable, so this scheme could lead to wrong polarity assignments for large variations in TS. Therefore, phase-sensitive T_1_-fitting, as previously proposed for inversion-recovery^25^, is performed based on phase-images.

Reported diastolic T_1_-times are in good agreement with a study on saturation-recovery at 3T^13^, which estimated diastolic T_1_-times of 1578 ± 42 ms and ECV-values of 0.20 ± 0.02. The present study found systolic SAPPHIRE T_1_ to be significantly shorter than diastolic SAPPHIRE T_1_, which agrees with previous studies using systolic MOLLI at 3T^8,26^. As one explanation, Kawel *et al*. presume a lower myocardial blood-volume concentration during systole. A potentially more dominant effect might be the reduction of partial-voluming, achieved by imaging at increased myocardial thickness. This might explain why no significant differences between systolic and diastolic T_1_ where found in other studies, where only small ROIs were drawn, thoroughly excluding borders and therefore partial-voluming^27^. Yet the comparison of diastolic and systolic MOLLI in the preliminary substudy of this work showed no difference. However, too small of a sample size might have induced lack of significance.

Systolic ECV was found to be significantly lower than diastolic ECV, which is as well in agreement with other publications at 3T^8,26^. Again, this might be due to less partial-voluming with the blood-pool, leading to lower native T_1_ and higher post-contrast T_1,_ conjointly resulting in a lower ECV for systole.

The present study has several limitations. A relatively small cohort of healthy volunteers, carefully selected to yield healthy myocardium with no age-related diffuse fibrosis, was recruited. Patients were not chosen from this strictly confined age-group and displayed various underlying pathologies, but other types of arrhythmia might also benefit from systolic acquisition. To facilitate integration into the clinical scan protocol, a single native mid-ventricular slice was acquired per patient, preventing segmentation according to the AHA-16-segment-model and ECV-estimation. In future work combination with slice-accelerated imaging will be explored to allow assessment of three left-ventricular slices in the scan protocol^28^. Furthermore, the proposed approach could be readily performed at 1.5T, which remains to be examined in future work.

In conclusion, our results show that the systolic SAPPHIRE saturation-recovery technique facilitates T_1_-mapping even in patients suffering from arrhythmia. Increased temporal resolution was achieved for the trade-off against slightly reduced precision. Systolic T_1_-mapping enabled imaging at increased myocardial thickness, resulting in significantly lower systolic T_1_-times and ECV-values. Hence, the proposed technique might be an alternative to diastolic T_1_-mapping, for clinical cohorts which displaying substantial variation in the RR-interval.
